# Leptin Levels in Acute and Recovered Eating Disorders: An Arm‐Based Network Meta‐Analysis

**DOI:** 10.1002/erv.3163

**Published:** 2024-12-06

**Authors:** Emanuele Cassioli, Lorenzo Lucherini Angeletti, Eleonora Rossi, Giulia Selvi, Elena Riccardi, Serena Siviglia, Roberta Buonanno, Valdo Ricca, Giovanni Castellini

**Affiliations:** ^1^ Psychiatry Unit Department of Health Sciences University of Florence Florence Italy

**Keywords:** anorexia nervosa, binge‐eating disorder, bulimia nervosa, leptin, network meta‐analysis

## Abstract

**Objective:**

This study aimed to provide a BMI‐adjusted meta‐analytical calculation of blood leptin levels across different eating disorders (EDs) including anorexia nervosa (AN), bulimia nervosa (BN), binge eating disorder (BED), recovered EDs, and healthy controls (HCs). The goal was to understand BMI‐independent leptin alterations and their potential as biomarkers.

**Method:**

PubMed and ClinicalTrials.gov were searched for studies reporting serum leptin in AN, BN, BED, or recovered EDs. A multilevel network meta‐analysis using a linear mixed‐effects meta‐regression model, adjusting for BMI, sex, and assay type, was performed on 146 studies (5048 patients, 3525 controls).

**Results:**

Significant differences in leptin levels were found across EDs. AN patients exhibited the lowest leptin levels, while BED patients had the highest. BN and recovered AN patients had leptin levels similar to AN, significantly lower than HCs. BMI, sex, and assay type were significant covariates. The model accounted for heterogeneity due to diagnostic criteria, assay types, and study‐level differences.

**Conclusions:**

Leptin levels in EDs are significantly altered beyond BMI effects, suggesting disease‐specific factors. These findings support leptin's potential as a biomarker for ED staging and prognosis. Further research is needed to explore leptin's role in ED pathogenesis and trajectory, to identify subpopulations and improve clinical interventions.

**Summary**

- This BMI‐adjusted arm‐based network meta‐analysis revealed significant leptin level differences across eating disorders, with BMI, sex, and assay type as significant covariate predictors.
- AN, BN and recovered AN patients exhibited lower leptin levels than healthy controls, while BED patients had the highest levels.
- Leptin levels in eating disorders are altered beyond BMI effects, suggesting potential as biomarkers for disease staging and prognosis.

## 1 Introduction

Eating disorders (EDs) are serious psychiatric illnesses characterised by a core psychopathology based on distorted cognitions about food, body shapes and weight, along with emotion dysregulation. These disorders involve dysfunctional eating behaviours and are associated with pathological weight fluctuations (American Psychiatric Association 2022). The main EDs diagnoses in adulthood are anorexia nervosa (AN), bulimia nervosa (BN), and binge eating disorder (BED) (American Psychiatric Association 2022). The severity and high prevalence worldwide of EDs, which is increasing (Galmiche et al. 2019; Silén and Keski‐Rahkonen 2022), explain the burden highlighted in terms of mortality, disability, quality of life and healthcare resources (Castellini et al. 2023; van Hoeken and Hoek 2020), especially combined with the unsatisfactory remission rates (Atwood and Friedman 2020; Steinhausen 2009) obtained with currently available treatments recommended by international guidelines (National Institute for Health and Care Excellence 2020). For this reason, in recent years, research has focused on identifying possible biological maintenance mechanisms, to improve the clinical characterisation of these diseases and potentially develop targeted interventions to facilitate their treatment.

In this context, increasing attention has been paid to the role of the adipokine leptin since its discovery in 1994 (Zhang et al. 1994). This hormone, produced by adipose tissue, positively correlates with Body Mass Index (BMI), with a role in regulating body energy homoeostasis through appetite modifications, modulation of physical activity, and general reward mechanisms (Oswal and Yeo 2010; Verhagen, Luijendijk, and Adan 2011). However, the underlying mechanisms are far from being comprehensively clarified (Flier and Maratos‐Flier 2017). Specifically, leptin can inhibit the reward response, with an originally adaptive mechanism that could become maladaptive in the context of an ED due to the particular meanings attributed to food and eating (Cassioli, Rossi, et al. 2020). In particular, given the fundamental role of hypoleptinemia in activating numerous adaptive physiological and behavioural changes in underweight individuals (Ahima et al. 1996; Hebebrand et al. 2022), it has been theorised that reaching very low levels of leptin can trigger a state of ‘entrapment’ in AN, characterised by what has recently been compared to an addiction to the state of starvation, with an exacerbation of all the components of ED‐specific psychopathology (Hebebrand, Plieger, et al. 2024). Consequently, recent studies have conceptualised leptin alteration in EDs not only as a consequence of body weight alterations, but as a maintenance factor of aberrant eating behaviours, and consequently of the disorder (Cassioli, Rossi, et al. 2020; Monteleone and Maj 2013). Therefore, in order to evaluate the interplay between leptin levels and the pathophysiology of EDs, as well as the treatment implications, it is necessary to clarify the variations in leptin across different EDs, accounting for various confounding factors, and their persistence after recovery.

While the most recent reviews and meta‐analyses available in the literature seem to suggest that blood leptin levels are reduced in AN and increased in BED compared to the population of healthy controls (HCs) without EDs (Baenas et al. 2023; Karageorgiou et al. 2020; Wu et al. 2024), it is not clear as of today whether these alterations are solely related to changes in BMI or if they can also be observed beyond what is expected based on body weight changes. Furthermore, data regarding BN are ambivalent (Monteleone and Maj 2013), and to date, there are no studies on large or aggregated samples that allow drawing significant conclusions on possible BMI‐independent alterations of leptin levels in this diagnosis. Finally, the question remains open regarding the restoration of normal leptin levels following remission from an ED. The literature data in this field are very scarce, and the few available studies (conducted mainly in the context of AN) report conflicting results without significant conclusions.

To date, no meta‐analytical calculations are available to establish the differences in leptin levels between the various EDs, recovered EDs, and HCs. The fact that most existing studies have focused on a single ED, and that very few studies have compared different diagnostic categories with each other, greatly reduces the number of direct comparisons available for meta‐analysis. Furthermore, the variability of existing laboratory methodologies for measuring leptin levels, and the availability of different procedures or commercial kits with different reference intervals, makes a meta‐analytical approach with traditional techniques particularly difficult.

Establishing whether there are alterations in basal leptin levels independent of BMI in the various EDs compared to normative levels appears fundamental to lay the foundations for an improvement in diagnostic tools and possibly therapeutic interventions targeted on biological maintenance mechanisms. Consequently, the objective of this study was to offer for the first time a BMI‐adjusted meta‐analytical calculation of blood leptin levels in AN, BN, BED, recovered EDs, and HCs, through a multilevel network meta‐analysis. This analysis approach would allow maximising the useable data in order to produce accurate estimates and multiple comparisons between all diagnostic groups, taking into account the heterogeneity deriving from the different laboratory assays and kits used.

## 2 Methods

The present network meta‐analysis was conducted and reported following the Preferred Reporting Items for Systematic Reviews and Meta‐Analyses (PRISMA) for network meta‐analyses extension guidelines (Hutton et al. 2015).

### 2.1 Search Strategy and Selection Criteria

A search was conducted on the PubMed and ClinicalTrials.gov databases. Regarding PubMed, the complete search string included field tags to exclude non‐original articles (i.e., reviews or meta‐analyses) or articles not written in English, and was as follows:

*leptin AND (eating disorder OR anorexia nervosa OR bulimia nervosa OR binge eating disorder) NOT review[pt] NOT systematic review[pt] NOT meta‐analysis[pt] AND eng[la]*

As for ClinicalTrials.gov, three separate searches were performed on the three diseases considered (AN, BN, and BED), each time entering the term ‘leptin’ as an additional search term. Only trials reported as ‘Completed’ or ‘Terminated’ were selected for screening. The final searches were conducted on April 5, 2024, and included all results from databases inception.

Inclusion criteria were as follows: written in English; provided data on human participants; provided original quantitative data; were not case reports or series; included patients with current or recovered clinical diagnoses of AN, BN or BED according to criteria compatible with the Diagnostic and Statistical Manual of Mental Disorders (DSM) or the International Classification of Diseases (ICD); provided measurements of serum leptin levels and average BMI for at least one diagnostic group; provided information regarding the laboratory methodology used for leptin measurement, and the procedure or commercial kit used.

Groups that included non‐remitted patients who, although still meeting the reference diagnostic criteria, were already receiving nutritional treatment and/or had already undergone any type of weight recovery were excluded. Healthy control groups were considered includable only if they did not present selection criteria based on the presence of pathologies or weight conditions (e.g., control groups selected based on the presence of obesity or constitutional thinness were not taken into consideration). These groups were excluded from the scope of the present meta‐analysis after an initial screening during the study protocol design phase, due to the extreme heterogeneity concerning these populations. Indeed, in most available studies, there is no clear definition of what is meant by constitutional thinness, reliable inclusion or exclusion criteria are not specified, and there is no adequate screening for subjects who previously met diagnostic criteria for EDs (and therefore might represent not so much controls as remitted EDs), subjects presenting concomitant conditions that could explain weight variation (e.g., metabolic or endocrine diseases, depressive or anxious pathology, emotional eating) or with subthreshold EDs (which could introduce significant bias).

Regarding the group of patients considered recovered, only studies that reported a precise definition of recovery compatible with the definitions of partial remission or full remission specified in the DSM‐5 (American Psychiatric Association 2022) were included. To be considered meta‐analysable, leptin values for each diagnostic group had to be reported as a mean and either standard deviation or standard error. In cases where a study included aggregated samples (e.g., patients with AN together with patients with BN, or patients with a current ED diagnosis together with recovered patients), it was included only if results related to at least one individual diagnostic group were reported. Articles were excluded if they were found to be duplicate reports of previously published studies. In cases where a study reported data regarding a sample that represented a subset of another study, only the report of the larger sample was included, regardless of the publication date.

### 2.2 Study Selection and Data Extraction

Titles and abstracts were screened independently by two teams (G.S., E.R. and L.A.L., S.S., R.B.). After the first screening phase, the same teams retrieved full reports of included records and further assessed them for eligibility. In cases of disagreement among reviewers, the full‐text report was retrieved and independently assessed and discussed by expert reviewers E.R. and E.C. A consensus was reached for all studies.

Finally, data extraction was conducted on eligible reports, retrieving the following information for each diagnostic group (AN, BN, BED, HCs, remitted ED): sample size, diagnostic criteria used for diagnosis, leptin levels (mean and either standard deviation or standard error), average BMI, and presence of men. Moreover, for each study, the laboratory methodology used for measuring leptin levels was extracted, as well as the name of the laboratory kit used. If commercial laboratory kits were not used, the laboratory methodology was still extracted along with a reference to the procedure used. In cases where a study reported data separately for two groups belonging to the same diagnosis (e.g., for the two subtypes of AN), all available data were extracted and included in the final analyses. Reports that did not include all the listed data were excluded from the final analyses.

Leptin measurements, when in different units of measure, were converted to nanogrammes per millilitre (ng/mL). Regarding the sex of the participants, preliminary screenings revealed a prevalence of data predominantly concerning female‐only samples. When a study did not include exclusion criteria based on sex, data relating to men and women were almost always presented in an aggregated manner (mixed‐sex sample), with an almost total absence of samples composed exclusively of men. Therefore, the data relating to the sex of patients in each diagnostic group was extracted using a dummy variable indicating the presence of a male or mixed‐sex sample, versus a women‐only sample.

### 2.3 Quality Assessment

Regarding the assessment of methodological quality, the adaptation for cross‐sectional studies of the Newcastle‐Ottawa Scale (NOS) was used (Modesti et al. 2016). Given the scope of the present study, the only relevant items were those related to sample selection (i.e., ED diagnosis) and assessment of leptin (i.e. the outcome). Moreover, in the present study, the raw blood leptin values were extracted from each included report, while the original outcomes of the reports or their statistical analyses were not of interest. For this reason, NOS items deemed irrelevant in the current meta‐analytical context were excluded, namely those concerning adequate sample size, comparability between responders and non‐responders, presence of precautions for confounding factors and additional variables, and appropriateness of statistical tests. All included studies received three points (out of three) regarding sample appropriateness since only reports with samples selected using validated criteria were included. Furthermore, all included studies received a score of two points (out of two) for outcome quality, as only reports that included laboratory leptin measurements describing the method and kit or procedure were included. Consequently, all selected studies received a score of five out of a maximum of five points and were therefore considered to be of high quality.

### 2.4 Statistics

Blood leptin levels for each diagnostic group were calculated with a multilevel arm‐based network meta‐analysis performed using a linear mixed‐effects meta‐regression model. Effect sizes were calculated as raw means. The model, which did not include an intercept in order to enhance interpretability, included the following moderators (fixed effects):

– ED diagnostic groups, coded as dichotomous dummy variables.
– BMI, as a variable centred on the overall mean. This was done to improve the interpretability of the fixed effects related to the diagnostic groups, which would otherwise have indicated the estimation of leptin values for a hypothetical patient with a BMI equal to zero. It is important to note that this variable was only centred, and not scaled, which means that the interpretation of the resulting fixed effect remains identical (i.e., the variation of leptin as BMI varies by one point).
– Men/Mixed‐sex Sample, as a dichotomous dummy variable indicating the presence of men within the sample (vs. a sample of only women), as a proxy for the difference in leptin levels between males and females.
– Assay type, a factor indicating the type of laboratory methodology used for the measurement of leptin, such as radioimmunoassay (RIA) or Enzyme‐Linked Immunosorbent Assays/Enzyme Immunoassays (ELISA/EIA). While this fixed effect only considered the type of biochemical assay, the model also accounted for specific individual kits and procedures (see below).

To further increase the accuracy of the model, several random effects were considered. First, in order to account for non‐independence arising from multiple effect sizes obtained in each study, the study‐level random effect was included. Secondly, an important source of heterogeneity is given by the diagnostic groups considered in each individual study: sampling from populations of different origins, the use of different inclusion and exclusion criteria, and other study‐specific factors make it necessary to include the diagnostic category as a random factor, nested within study. This approach provides a more flexible and realistic model of the data, as it acknowledges the potential for study‐level differences in the relationship between ED diagnoses and leptin levels. For the same reason, a random effect was also added for the ED diagnostic criteria that were used in each study. Finally, a random effect was specified for the leptin measurement assay kit or procedure, to account for the potential correlation among effect sizes that shared the same measurement methods. This effect was included to mitigate the possible bias arising from technical variations between methods, potentially also related to the methodological progress observed over the last 3 decades.

To test for residual heterogeneity, the *Q*_*E*_‐test was performed, whereas the *Q*_*M*_‐test was used as an omnibus test of moderators. The heterogeneity statistic commonly used in meta‐analyses, *I* ^*2*^, was computed overall and separately for each random effect considered, according to the generalised method for multilevel meta‐analytic meta‐regression models illustrated by Nakagawa and Santos (Nakagawa and Santos 2012).

Following the principles of network meta‐analyses, a league table was ultimately produced with contrasts between all the diagnostic groups considered, in order to allow direct comparisons regarding leptin levels even in the absence of studies that explicitly reported such analyses.

All analyses were performed using R statistical software v4.3.3 (R Core Team 2024), and the following libraries: dplyr, forestploter, ggplot2, metafor, and multcomp (Dayimu 2023; Hothorn, Bretz, and Westfall 2008; Viechtbauer 2010; Wickham 2016; Wickham et al. 2021).

## 3 Results

### 3.1 Characteristics of the Included Studies

**FIGURE 1:**
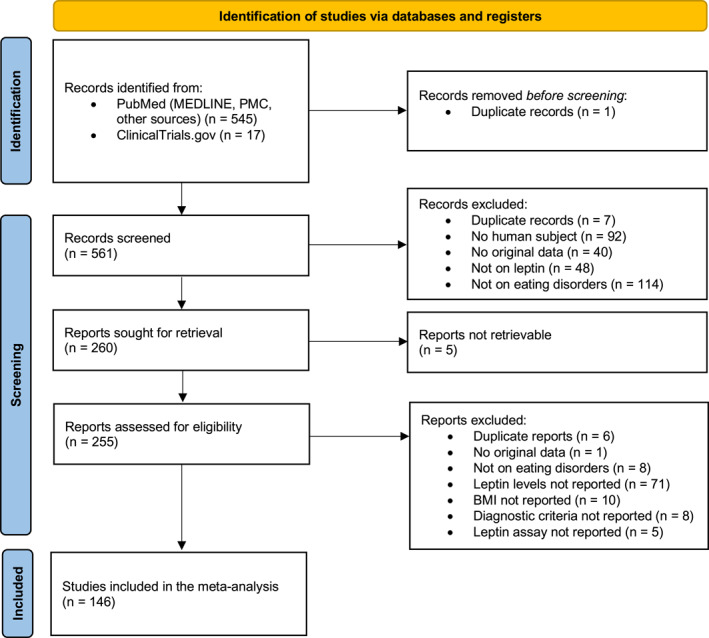
Preferred Reporting Items for Systematic reviews and Meta‐Analyses (PRISMA) flow diagram of study selection process.

**FIGURE 2:**
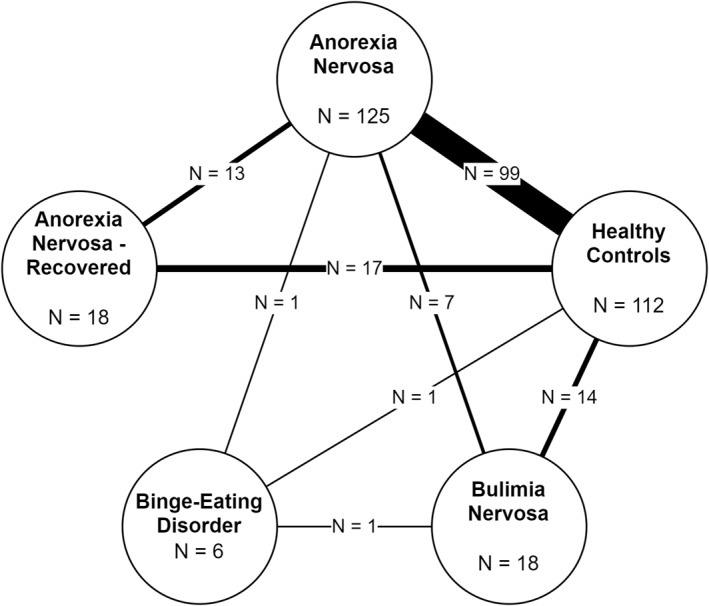
Network diagram of study comparisons, illustrating the comparative studies of blood leptin levels across different eating disorders and healthy controls included in the meta‐analysis. Each node shows the total number of studies reporting leptin levels for a diagnostic group. The lines between nodes indicate direct comparisons between groups, with the thickness of each line proportional to the number of studies comparing those groups.

A total of 562 records were identified from the sources and screened for eligibility in the present study. Figure 1 shows the flow diagram. Of the 260 records initially selected for full‐text screening, it was not possible to obtain the report for five of them. All these five records corresponded to entries on ClinicalTrials.gov related to studies for which no results were published and for which no publication could be traced. A total of 146 studies were included in the final analysis (Table S1). Among all studies, the vast majority reported leptin measurements in patients with acute AN (*n* = 125), whereas only few investigated patients with BN (*n* = 18) and BED (*n* = 6), and 18 measured leptin levels in patients with remitted AN. No studies were included that reported blood leptin levels in patients with remitted BN or BED. A comparison with HCs was reported in 112 studies. Figure 2 illustrates the network of direct comparisons that could be extracted from the included studies. Most studies have reported comparisons with HCs, while the available data on direct comparisons between different EDs are very limited, particularly regarding BN and BED (Figure 2).

Regarding the laboratory methodology used for measuring blood leptin levels, most studies have used radioimmunoassays (RIA, *n* = 83), while the remaining studies have used Enzyme‐Linked Immunosorbent Assays/Enzyme Immunoassays (ELISA/EIA, *n* = 62), apart from a single study that employed a Ligand‐Mediated Immunofunctional Assay (LIFA). In total, 38 different commercial kits or procedures were used for measuring leptin levels across all reports.

### 3.2 Meta‐Analysis

**FIGURE 3:**
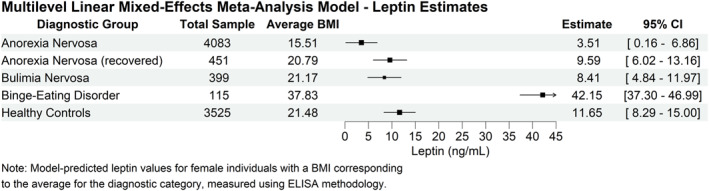
Forest plot of model‐based leptin estimates. Each entry shows the total sample size, average body mass index (BMI, in kg/m^2^), leptin estimate (in ng/mL), and 95% confidence interval (CI) for each group. The horizontal lines represent the 95% CIs for each estimate.

A multilevel meta‐analysis model was computed on 302 blood leptin values extracted from the included studies, for a total of 5048 patients and 3525 HCs. Mean leptin levels were calculated for each diagnostic group, adjusting for BMI, presence of mixed‐sex samples and laboratory methodology used. The results regarding fixed effects estimates are reported in Table 1. Figure 3 shows the model‐predicted values for each diagnosis, for a measurement on a woman using the ELISA/EIA methodology with an average BMI for the corresponding group.

**TABLE 1:** Multilevel linear mixed‐effects meta‐analysis model for blood leptin levels (in ng/mL) for different eating disorders.

Fixed effect	Estimate	Standard error	*p*‐value	95% CI lower bound	95% CI upper bound
Anorexia nervosa	6.31	1.74	< 0.001	2.89	9.73
Anorexia nervosa (recovered)	8.04	1.83	< 0.001	4.45	11.63
Binge‐eating disorder	26.57	3.01	< 0.001	20.66	32.47
Bulimia nervosa	6.54	1.83	< 0.001	2.95	10.14
Healthy controls	9.53	1.73	< 0.001	6.13	12.93
Body Mass index (centred)	0.82	0.10	< 0.001	0.62	1.02
Mixed‐sex sample (vs. women‐only)	−1.77	0.27	< 0.001	−2.30	−1.23
Assay type LIFA (vs. ELISA)	−0.75	3.13	0.810	−6.89	5.39
Assay type RIA (vs. ELISA)	1.33	0.57	0.020	0.22	2.44

*Note:* Test for Residual Heterogeneity: *Q*_*E*_(df = 298) = 6038.4, *p* < 0.001. Test of Moderators: *Q*_*M*_(df = 9) = 1068.63, *p* < 0.001.

Abbreviations: CI, confidence interval; ELISA, enzyme‐linked immunosorbent assay; LIFA, ligand‐mediated immunofunctional assay; RIA, radioimmunoassay.

**FIGURE 4:**
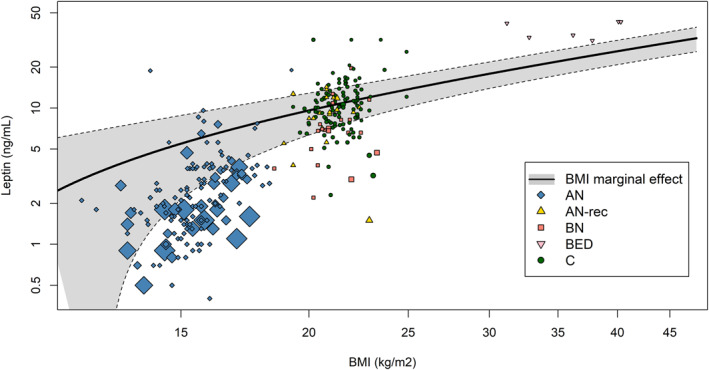
Meta‐regression analysis of leptin levels (in ng/mL) as a function of Body Mass Index (BMI, in kg/m^2^) across different eating disorder groups and healthy controls. Different symbols denote the various groups, with sizes proportional to the sample size. The solid black line represents the marginal effect of BMI on leptin levels, with the shaded area indicating the 95% confidence interval. Both axes are on a logarithmic scale.

All estimates of mean blood leptin values in different diagnoses were statistically significant (*p* < 0.001). The coefficient related to BMI was also statistically significant (Table 1), indicating a positive association between BMI and leptin levels. Specifically, an average increase of 0.82 ng/mL in leptin was calculated for each additional BMI point (Table 1). The BMI marginal effect was illustrated in Figure 4, which also includes a bubble plot of all extracted leptin levels and related BMI values, grouped by diagnoses. BMI‐independent diagnosis effects are also visible in Figure 4, as the patient groups tend to show leptin levels above or below the computed line. The model also confirmed the presence of a difference between sexes, with lower leptin levels in samples composed entirely or partially of men, compared to samples of only women (−1.77 ng/mL, Table 1). There was a significant difference between ELISA/EIA and RIA assay types, with the latter measuring higher levels of leptin on average (Table 1).

The omnibus *Q*_*M*_‐test of moderators indicated that the variables included in the model significantly explained the heterogeneity observed in the included studies, although the significant *Q*_*E*_‐test for residual heterogeneity indicated the existence of variability in the data not fully explained by the moderators included in the present model alone (Table 1). The calculated *I* ^*2*^ statistic was 99.7%, indicating that almost all the variance was attributable to heterogeneity. Specifically, most of the variance was explainable by the heterogeneity introduced by the different ED criteria used for diagnosis (66.0%), while the remainder was attributable to different kits or procedures used for the measurement of leptin (11.4%), study‐level heterogeneity (5.4%) and related to diagnostic groups (17.0%).

**FIGURE 5:**
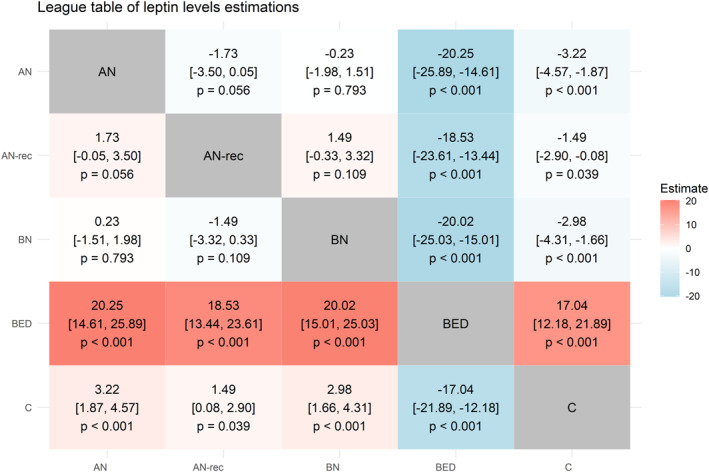
League table comparing leptin level estimations across different eating disorder groups and healthy controls. Each cell in the table shows the estimated difference in leptin levels (ng/mL) between two groups, along with the 95% confidence interval (CI) and *p*‐value for the comparison. The colour gradient indicates the magnitude and direction of the leptin level differences, with red shades representing higher estimates and blue shades representing lower estimates.

Finally, multiple comparisons were performed between the various diagnostic groups, and the results were included in the league table shown in Figure 5. After adjusting for the effect of BMI, sex and assay type, the diagnosis of AN was associated with the lowest leptin values, while the highest were in patients with BED (Figure 5). The levels measured in BN, which were similar to those found in AN, were significantly lower than in HCs (Figure 5). As for patients with AN in remission, their leptin levels were not different than those of acute‐phase AN patients, while remaining lower than in healthy subjects (Figure 5).

## 4 Discussion

This study offered for the first time a meta‐analytic calculation of basal blood leptin values in EDs, comparing them between the different diagnostic categories, recovered AN, and HCs. The results firstly confirmed that, even after adjusting for the average BMI of the samples considered, leptin levels are reduced in AN and increased in BED, compared to control groups. As for BN, a BMI‐independent pathological reduction in leptin levels compared to HCs was confirmed, with no significant difference from AN. This is the first meta‐analysis that considered AN, BN, and BED together, comparing leptin levels with those of HCs, individuals recovered from AN, and various diagnostic categories with each other. Compared to the previously published meta‐analyses on this topic, which focused exclusively on AN (Karageorgiou et al. 2020; Wu et al. 2024), this study also provides an innovative multivariate meta‐regression model with estimates of leptin values for each group, adjusted for various confounding factors in addition to BMI, including sex, laboratory methodology, the kit used, and diagnostic criteria.

The alterations of blood leptin levels in AN and BED have been known for a long time (Baenas et al. 2023; Karageorgiou et al. 2020; Wu et al. 2024), and this BMI‐adjusted meta‐analysis confirmed that this phenomenon goes beyond what can be predicted based on weight alone. To explain these alterations, different mechanisms can be hypothesised depending on the disease. Regarding AN, previous comparisons with underweight individuals without AN (constitutional thinness) confirmed that the latter had leptin levels that, although reduced in line with the reduction in BMI, were still significantly higher than those observed in AN (Bailly et al. 2021; Karageorgiou et al. 2020). Various mechanisms can be hypothesised for this difference, ranging from different body composition to the presence of malnutrition and alteration of the circadian rhythm, to the possibility of a role played by prolonged exposure to psychological distress (Bailly et al. 2021; Bouillon‐Minois et al. 2021; Germain et al. 2007).

Regarding BED, the main hypotheses raised in the existing literature include a role in the pathology‐specific elevation of leptin by binge eating episodes (consequently to the transient positive energy balance), different eating patterns, and mechanisms of leptin resistance (Adami et al. 2002; Miller et al. 2014; Monteleone et al. 2000).

The results in the BN group were consistent with many of the studies and literature reviews that had shown reduced leptin levels in this group (Jimerson et al. 2000 2010; Monteleone 2002; Monteleone and Maj 2013). Such alterations, in the presence of a non‐underweight BMI, could still be related to the underlying malnutrition, with consequent alterations in body composition (Lopes et al. 2023; Vaz, Guisado, and Peñas‐Lledó 2003). Moreover, the absence of a statistically significant difference compared to AN in the between‐group comparisons is compatible with the high rate of diagnostic crossover highlighted between these two diagnoses in the course of the illness (Castellini et al. 2011), confirming the considerable overlap between the two pathologies, also in terms of biological characteristics. However, these findings related to BN should be interpreted with caution because it is entirely possible that, given the known limitations of the current nosography on EDs, there may be subcategories of the disorder that are not adequately considered. In fact, other studies report leptin values in BN that are not dissimilar to those of controls (Monteleone and Maj 2013). The study by Monteleone (2002) provides further data that help clarify this possible ambiguity: in particular, two groups of patients with BN were identified, one with decreased leptin values and one with normal values (Monteleone 2002); the further differences between the clinical characteristics of these two groups and correlational results led the Authors to hypothesise the chronicity of the disease and the frequency of binge‐purging behaviours as the cause of leptin alterations, primarily or as a proxy for latent malnutrition (Monteleone 2002).

Alternative hypotheses could be considered in light of the interaction of leptin with other energy‐regulating hormones, such as ghrelin. Ghrelin has been recently conceptualised as a stress hormone (Stone, Harmatz, and Goosens 2020), with studies on traumatised populations reporting a chronic alteration of the ghrelin axis (Malik et al. 2020; Yousufzai et al. 2018). Furthermore, recent data have confirmed an association between childhood traumatic experiences and increased blood levels of ghrelin in patients with EDs (Rossi et al. 2021). Given the inhibitory power of ghrelin on endogenous leptin secretion (Haass‐Koffler et al. 2015), it is conceivable that in individuals with a history of childhood trauma, the increase in ghrelin levels contributes, along with the other factors described above, to the decrease in leptin. From this perspective, leptin, like ghrelin, could represent a biological marker of the so‐called ‘maltreated eco‐phenotype’ of EDs, a subpopulation of patients with a history of childhood abuse and distinct psychopathological features (Rossi et al. 2024). The associations between reduced leptin levels and higher frequencies of binge‐purging behaviours and comorbidity with borderline personality disorder (Atmaca et al. 2002; Monteleone 2002), both characteristics highlighted in the maltreated eco‐phenotype (Ball and Links 2009; Rossi et al. 2024), corroborate this hypothesis. According to these observations, the greater heterogeneity of leptin findings in BN could therefore be explained in light of different prevalences of early adverse experiences in the different samples.

Finally, the present data indicated the persistence of lower leptin levels compared to controls even in the group of individuals previously affected by AN and considered recovered, with values in fact comparable to those observed in the group of patients with BN. This result may seem apparently in contrast with studies that have shown an increase in leptin in weight‐recovered subjects with AN (Hebebrand et al. 1997; Holtkamp et al. 2003; Lob et al. 2003). However, it is important to note that in the present study, regarding the analyses on recovered individuals, only articles that considered remission criteria compatible with those of the DSM‐5 were included. Indeed, in adult AN, these criteria require at least the recovery of normal weight for a sustained period of time (American Psychiatric Association 2022). This led to the exclusion of all reports that evaluated leptin levels after a fixed follow‐up period from the start of treatment, or after a partial weight recovery or one that was not maintained over time. This is relevant because the peak of leptin elevation in patients with AN appears to be a phenomenon that is observed mainly transiently during the weight recovery phase (Hebebrand et al. 1997), while the objective of the present study was to highlight the leptin levels at which patients stabilise once remission is obtained.

Various interpretations of this finding are possible. On one hand, what has been described above for BN is potentially applicable to recovered AN as well. In particular, high prevalences of childhood trauma are also evident in AN (Longo et al. 2019; Molendijk et al. 2017), which could be associated with the same aforementioned trait alterations resulting in dysregulation of the leptinergic axis. Moreover, beyond the presence of childhood trauma, previous studies have shown chronic elevations of plasma ghrelin levels in patients recovered from AN even many years later (Bernardoni et al. 2020; Holsen et al. 2014), with possible repercussions on leptin levels as well. Recent data have also highlighted an association between a genetic predisposition for lower levels of leptin and the risk of developing AN (Peters et al. 2021). This trait characteristic would therefore be consistent with the persistence of hypoleptinemia after remission, and could contribute to the slowness in recovery and the subsequent risk of relapse (Peters et al. 2021). Given the potentially long‐lasting effect of fasting and caloric restriction on the reduction of circulating leptin with receptor sensitisation (Mendoza‐Herrera et al. 2021; Varkaneh Kord et al. 2021), it is also conceivable that the rigid dietary regimen maintained for a long time by AN patients could induce persistent alterations in the leptinergic system.

Finally, it is possible that aberrant eating behaviours promoting the reduction of leptin may persist even in a subject considered to be in remission. It is well known that many patients with remitted AN maintain levels of ED‐related psychopathology higher than HCs (Tomba et al. 2019). Consequently, it can be hypothesised that subthreshold restrictive behaviours could lead to a state of latent malnutrition with associated alterations in leptin levels. Moreover, these residual symptoms together with the orthorexic tendencies often observed in remitted AN after treatment (Segura‐Garcia et al. 2015) could lead to following low‐carbohydrate and protein‐rich diets. In turn, these persistent dysfunctional dieting behaviours are associated with a reduction in circulating leptin levels possibly correlated with a sensitisation of the leptinergic system (Weigle et al. 2005). If these hypotheses were confirmed, monitoring leptin levels post‐treatment would allow distinguishing ‘truly’ recovered individuals from those who, despite meeting the commonly used criteria for the definition of remission, maintain a subthreshold ED‐related psychopathology. Indeed, in post‐treatment patients with AN low leptin levels have been associated with the lack of resumption of menses (Brambilla et al. 2003), a phenomenon which, in turn, is considered a marker of recovery for many patients, although not all (Castellini et al. 2020). Thus, leptin alteration might also represent a candidate biomarker for staging the recovery process in EDs.

The results of this study should be interpreted in light of some limitations. First, although the inclusion criteria were as broad as possible, some diagnostic subgroups were still underrepresented. This was particularly evident for the BED group, in which only six studies were available. While currently there are no defined guidelines on the minimum number of studies to include in a network meta‐analysis to ensure that the results are sufficiently robust, a systematic review conducted on this type of investigation highlighted that among the 248 network meta‐analyses considered, the median number of total individual studies (including all arms) from which data were extracted was 10, with a median number of arms equal to 5 (Greco et al. 2015). The present study included 146 individual reports for 5 arms, and the arm with the fewest studies (i.e., BED) comprised 6 studies. Therefore, it would be positioned in the higher percentiles in terms of data availability for estimating effect sizes (Greco et al. 2015). Additionally, the computed confidence intervals depend on sample size, and despite the larger standard error regarding the estimates for the BED group (visible in Table 1), significant statistical tests indicate reliable results within the selected Type I error threshold (*α* = 0.05). This, combined with the fact that the results on BED are consistent with all previous studies, should make the results sufficiently robust. Furthermore, it was not possible to obtain meta‐analysable data regarding patients with a history of BN or BED in recovery. Moreover, different illness trajectories (e.g., initial onset of AN and later diagnostic crossover to another ED vs. persistence of a single diagnosis) might also account for specific leptin levels changes across time, due to psychopathological, behavioural and weight fluctuations. In the present study, this aspect could not be taken into consideration, mainly due to the very limited availability of data (most studies do not report this information). Future studies could explore the role of different illness histories in determining possible changes in leptin levels. Regarding the analysis methodology, all models were based on aggregate data, like most meta‐analytic analyses (Tudur Smith et al. 2016), due to the unavailability of the original datasets needed for Individual Participant Data (IPD) analyses. Although the two methods generally provide consistent results (Tudur Smith et al. 2016), future studies are needed to confirm the present findings with IPD‐based analyses.

The present meta‐analysis also had numerous strengths. Firstly, the design as an arm‐based network meta‐analysis allowed the inclusion of all studies reporting leptin measurements in EDs, which was not possible in previous meta‐analyses that only included direct comparisons with controls (Karageorgiou et al. 2020; Wu et al. 2024), and for the first time permitted the comparison of all EDs with each other, as well as with HCs and recovered AN patients. Additionally, the complex meta‐regression model included various fixed and random effects, which allowed for more accurate estimates and comparisons directly in the native unit of measurement (ng/mL), avoiding meta‐analytic measures like the standardised mean difference that often have less clinical interpretability. This allowed for the inclusion of several hundred more patients compared to previous meta‐analysis (Karageorgiou et al. 2020; Wu et al. 2024), while also highlighting a BMI effect in the meta‐regression that previously had not reached statistical significance (Karageorgiou et al. 2020). Finally, the design included the type of procedure or kit as a random correction factor in the mixed model. This represents one of the major strengths of the study and a novel approach compared to previous studies available in the literature. However, despite the methodological and statistical precautions implemented, the results still showed high levels of residual heterogeneity, suggesting that there are numerous other factors influencing leptin levels in patients with EDs that were not considered in the present study.

### 4.1 Conclusions

In conclusion, the present meta‐analysis shed light on the leptin alterations highlighted in the literature in patients with EDs. Our results indicated that leptin levels may have significant correlates beyond the alterations in BMI, potentially linked to psychopathological factors associated with EDs. Given these general considerations, leptin represents a promising biomarker in the context of a possible staging model of EDs. A possible clinical application might be monitoring the refeeding phase in AN to personalise dietary interventions, in order to minimise the leptin peak observed during the weight recovery phase (Hebebrand et al. 1997). Since this peak is possibly associated with an increased risk of relapse (Holtkamp et al. 2004), these additional interventions could significantly improve prognosis and facilitate weight recovery in this phase of the illness. Although a study on underage patients with AN did not show a predictive power of post‐refeeding leptin levels in relation to BMI 1 year later, it is important to note that many potential confounding effects were present (including a non‐fixed follow‐up length after discharge), and the result was confirmed in a secondary analysis after simplifying the model with the removal of a non‐significant covariate (Seitz et al. 2016). Therefore, studies with larger samples in adult populations are needed, carefully taking into account the possible various confounding effects.

Furthermore, in all EDs, it could be useful to evaluate BMI‐adjusted leptin levels as markers of the persistence of subclinical elements of the disorder to be addressed in clinical and psychotherapeutic contexts, such as fasting, compensatory exercise, or strict diets in AN or BN, and binge‐eating in BED.

However, further studies are needed to improve the understanding of the role of these alterations in the pathogenesis and trajectory of EDs. Specifically, future research should focus on identifying factors characterising specific subpopulations, such as those with a history of childhood trauma, and employing more in‐depth instrumental investigations like bioimpedance analysis for a more accurate examination of body composition. Furthermore, it is of primary importance to make comparisons with populations that present variations in body weight in the absence of an ED (e.g. constitutional low weight, obesity without BED), collecting data in a rigorous way, e.g. with exclusion criteria that include screening and diagnostic procedures for current or past EDs, even subthreshold ones, or concomitant conditions that could explain weight variation (e.g., metabolic or endocrine diseases, depressive or anxious pathology, emotional eating).

If a trait alteration of leptin pre‐existing the overt onset of the disorder is confirmed, it could have significant applications in screening as a potential diagnostic marker and for better clinical characterisation of EDs. Conversely, if leptin alterations are found to be state‐dependent, influenced by aberrant eating behaviours, their assessment could aid clinicians in monitoring therapeutic progress and identifying residual symptoms post‐treatment. This would allow for better stratification of relapse risk and the development of early screening procedures.

Moreover, leptin could help identify different subpopulations within ED patients, such as those with a history of abuse versus those without, and distinguish between different stages of the disease, such as chronic versus non‐chronic and remitted versus non‐truly remitted. This differentiation could lead to more tailored prognoses and targeted interventions.

Preliminary studies suggest the potential effectiveness of leptin substitution therapy in aiding weight restoration and reducing aberrant eating behaviours or, in some cases, rumination on food‐related issues (Hebebrand, Antel, et al. 2024; Hebebrand, Hinney, and Antel 2023; Milos et al. 2020; Wronski et al. 2023). However, to date, the available studies are predominantly uncontrolled case series, involving small samples, lacking placebo comparisons, and long‐term follow‐up. Additionally, it is well‐known that the core psychopathological dimensions characterising EDs, such as body image disturbance and fear of weight gain, are generally resistant to pharmacological treatments (Cassioli, Sensi, et al. 2020) and are better addressed through psychotherapeutic interventions, as recommended by major international guidelines (Crone et al. 2023; National Institute for Health and Care Excellence 2020). Therefore, to explore the potential future application of leptin treatments in patients with EDs, further studies are required to evaluate its role as a moderator and mediator of treatment efficacy, including psychotherapy outcomes, a gap that current research has yet to address.

## Conflicts of Interest

The authors declare no conflicts of interest.

## Supporting information

Supporting Information S1

## Data Availability

The data that support the findings of this study are available from the corresponding author upon reasonable request.
