# Supplementary material for: Leptin Levels in Acute and Recovered Eating Disorders: An Arm‐Based Network Meta‐Analysis
Source: Eur Eat Disord Rev. 2024 Dec 6;33(3):525–37. doi: 10.1002/erv.3163 (PMC11965547; doi:10.1002/erv.3163)
Supplement: Supplementary file 1 — Supporting Information S1 [file ERV-33-525-s001.docx]

# Supplementary Table 1 – Characteristics of included studies

| Study | PubMed IDentifier (PMID) | ED Criteria | ED Samples | Control Samples | Leptin Assay Type |
| --- | --- | --- | --- | --- | --- |
| Grinspoon, 1996^1^ | 8923829 | DSM-IV | AN (n = 22) | HCs (n = 23) | RIA |
| Baranowska, 1997^2^ | 9439531 | Feighner et al. (1972) and Russell (1979) | AN (n = 15) | HCs (n = 19) | RIA |
| Casanueva, 1997^3^ | 9169091 | DSM-IV | AN (n = 10) | HCs (n = 18) | RIA |
| Karhunen, 1997^4^ | 9138042 | DSM-IV | BED (n = 11) |  | RIA |
| Mantzoros, 1997^5^ | 9177394 | DSM-III | AN (n = 11) | HCs (n = 15) | RIA |
| Audi, 1998^6^ | 9857982 | DSM-IV | AN (n = 27) AN-rec (n = 17) |  | RIA |
| Balligand, 1998^7^ | 9578509 | DSM-III-R | AN (n = 29) | HCs (n = 80) | RIA |
| Eckert, 1998^8^ | 9506729 | DSM-IV | AN (n = 29) | HCs (n = 14) | RIA |
| Herpertz, 1998^9^ | 9838240 | DSM-IV | AN (n = 5) | HCs (n = 6) | RIA |
| Støving, 1998^10^ | 9713566 | DSM-IV | AN (n = 18) | HCs (n = 11) | RIA |
| Ballauff, 1999^11^ | 10089013 | DSM-IV | AN (n = 16) |  | RIA |
| Gendall, 1999^12^ | 10418705 | DSM-III-R | AN-rec (n = 23) | HCs (n = 31) | RIA |
| Haluzik, 1999a^13^ | 10575656 | DSM-IV | AN (n = 17) | HCs (n = 17) | ELISA |
| Haluzik, 1999b^14^ | 10523055 | DSM-IV | AN (n = 11) | HCs (n = 11) | RIA |
| Lear, 1999^15^ | 10441243 | DSM-IV | AN (n = 15) | HCs (n = 16) | RIA |
| Morgan, 1999^16^ | 10372743 | DSM-IV | AN (n = 7) |  | RIA |
| Nakai, 1999^17^ | 10199758 | DSM-IV | AN (n = 20) | HCs (n = 20) | RIA |
| Soyka, 1999^18^ | 10599707 | DSM-IV | AN (n = 11) | HCs (n = 15) | RIA |
| Støving, 1999^19^ | 10372710 | DSM-IV | AN (n = 8) | HCs (n = 11) | RIA |
| Baranowska, 2000^20^ | 11220530 | Feighner et al. (1972) and Russell (1979) | AN (n = 30) | HCs (n = 25) | RIA |
| Brewerton, 2000^21^ | 10938446 | DSM-III-R | BN (n = 27) | HCs (n = 16) | RIA |
| Monteleone, 2000^22^ | 10902800 | DSM-IV | BN (n = 12) | HCs (n = 10) | RIA |
| Nedvidkova, 2000^23^ | 10921449 | DSM-IV | AN (n = 15) | HCs (n = 15) | RIA |
| Polito, 2000^24^ | 10837290 | DSM-IV | AN (n = 16) AN-rec (n = 14) | HCs (n = 22) | RIA |
| Baranowska, 2001^25^ | 11600878 | DSM-IV | AN (n = 19) BN (n = 13) | HCs (n = 19) | RIA |
| Adami, 2002a^26^ | 12119579 | Spitzer et al. (1993) | BED (n = 14) |  | RIA |
| Adami, 2002b^27^ | 11890967 | Spitzer et al. (1993) | BED (n = 22) |  | RIA |
| Heer, 2002^28^ | 11978602 | DSM-IV | AN (n = 19) |  | RIA |
| Krizova, 2002^29^ | 12489569 | DSM-IV | AN (n = 15) | HCs (n = 15) | ELISA |
| Monteleone, 2002a^30^ | 12461192 | DSM-IV | AN (n = 33) BN (n = 56) | HCs (n = 38) | ELISA |
| Monteleone, 2002b^31^ | 12140788 | DSM-IV | BN (n = 45) |  | ELISA |
| Støving, 2002^32^ | 11940052 | DSM-IV | AN (n = 13) | HCs (n = 10) | RIA |
| Brambilla, 2003^33^ | 12834819 | DSM-IV | AN-rec (n = 22) | HCs (n = 20) | ELISA |
| Brown, 2003^34^ | 12647266 | DSM-III-R | AN-rec (n = 18) | HCs (n = 18) | RIA |
| Calandra, 2003^35^ | 12880190 | DSM-IV | AN (n = 12) | HCs (n = 12) | ELISA |
| Delporte, 2003^36^ | 12519408 | DSM-IV | AN (n = 26) | HCs (n = 24) | RIA |
| Holtkamp, 2003a^37^ | 14602745 | DSM-IV | AN (n = 88) |  | RIA |
| Holtkamp, 2003b^38^ | 12842170 | DSM-IV | AN (n = 18) | HCs (n = 18) | RIA |
| Krsek, 2003^39^ | 14649784 | DSM-IV | AN (n = 16) | HCs (n = 13) | ELISA |
| Lindberg, 2003^40^ | 12707489 | DSM-IV | AN (n = 11) | HCs (n = 11) | RIA |
| Lob, 2003^41^ | 12951634 | DSM-IV | AN (n = 20) |  | LIFA |
| Misra, 2003^42^ | 12915674 | DSM-IV-TR | AN (n = 43) | HCs (n = 38) | RIA |
| Pannacciulli, 2003^43^ | 12679468 | DSM-IV | AN (n = 11) | HCs (n = 26) | RIA |
| Weinbrenner, 2003^44^ | 14506487 | DSM-IV | AN (n = 51) | HCs (n = 51) | ELISA |
| Holtkamp, 2004^45^ | 15110928 | DSM-IV | AN (n = 18) | HCs (n = 18) | RIA |
| Miller, 2004^46^ | 15356043 | DSM-IV | AN (n = 116) |  | RIA |
| Misra, 2004^47^ | 15240636 | DSM-IV | AN (n = 23) | HCs (n = 21) | RIA |
| Tagami, 2004^48^ | 15070952 | DSM-IV | AN (n = 31) BN (n = 11) | HCs (n = 16) | RIA |
| Bosy-Westphal, 2005^49^ | 15793670 | DSM-IV | AN (n = 23) | HCs (n = 43) | RIA |
| Dostálová, 2005^50^ | 15721489 | DSM-IV | AN (n = 9) | HCs (n = 11) | RIA |
| Haas, 2005^51^ | 15817868 | DSM-IV | AN (n = 57) | HCs (n = 49) | RIA |
| Housova, 2005^52^ | 15598689 | DSM-IV | AN (n = 26) BN (n = 15) | HCs (n = 12) | ELISA |
| Manara, 2005^53^ | 16755167 | DSM-IV | AN (n = 24) BN (n = 24) |  | RIA |
| Monteleone, 2005^54^ | 15841111 | DSM-IV | AN (n = 15) BN (n = 12) BED (n = 11) | HCs (n = 15) | ELISA |
| Oświecimska, 2005^55^ | 16136022 | DSM-IV | AN (n = 19) |  | RIA |
| Shimizu, 2005^56^ | 15771692 | DSM-IV | AN (n = 12) |  | RIA |
| Bossu, 2006^57^ | 16912058 | DSM-IV | AN (n = 6) | HCs (n = 7) | RIA |
| Dostálová, 2006^58^ | 16889937 | DSM-IV | AN (n = 13) | HCs (n = 16) | RIA |
| Holtkamp, 2006^59^ | 16376860 | DSM-IV | AN (n = 26) |  | RIA |
| Miljic, 2006^60^ | 16449333 | DSM-IV | AN (n = 9) |  | RIA |
| Misra, 2006a^61^ | 17023694 | DSM-IV | AN (n = 39) | HCs (n = 39) | RIA |
| Misra, 2006b^62^ | 16549537 | DSM-IV | AN (n = 35) |  | RIA |
| Tanaka, 2006^63^ | 16643914 | DSM-IV | BN (n = 24) | HCs (n = 25) | ELISA |
| Brambilla, 2007^64^ | 17395395 | DSM-IV | AN (n = 20) | HCs (n = 10) | ELISA |
| Dolezalova, 2007^65^ | 17953628 | DSM-IV | AN (n = 12) | HCs (n = 18) | ELISA |
| Dostálová, 2007a^66^ | 17379007 | DSM-IV | AN (n = 10) | HCs (n = 15) | RIA |
| Dostálová, 2007b^67^ | 17184143 | DSM-IV | AN (n = 10) | HCs (n = 12) | RIA |
| Germain, 2007^68^ | 17413094 | DSM-IV | AN (n = 12) | HCs (n = 7) | RIA |
| Ohwada, 2007^69^ | 17998762 | DSM-IV | AN (n = 26) | HCs (n = 7) | RIA |
| Dostálová, 2008^70^ | 18559909 | DSM-IV | AN (n = 17) | HCs (n = 17) | ELISA |
| Dynesen, 2008^71^ | 18175732 | DSM-IV | BN (n = 19) | HCs (n = 20) | ELISA |
| Ehrlich, 2008a^72^ | 19067259 | DSM-IV | AN (n = 59) AN-rec (n = 35) | HCs (n = 59) | ELISA |
| Ehrlich, 2008b^73^ | 18423888 | DSM-IV | AN (n = 34) AN-rec (n = 19) | HCs (n = 35) | ELISA |
| Ehrlich, 2008c^74^ | 18317683 | DSM-IV | AN (n = 43) | HCs (n = 49) | ELISA |
| Guerdjikova, 2008^75^ | 18058852 | DSM-IV | BED (n = 44) |  | RIA |
| Holtkamp, 2008^76^ | 18385926 | DSM-IV | AN (n = 28) | HCs (n = 19) | RIA |
| Misra, 2008a^77^ | 19064506 | DSM-IV | AN (n = 15) | HCs (n = 15) | RIA |
| Misra, 2008b^78^ | 18089697 | DSM-IV | AN (n = 34) | HCs (n = 33) | RIA |
| Nakahara, 2008^79^ | 17919459 | DSM-IV | AN (n = 11) | HCs (n = 11) | RIA |
| Dostálová, 2009^80^ | 19093738 | DSM-IV | AN (n = 10) BN (n = 10) | HCs (n = 20) | ELISA |
| Ehrlich, 2009a^81^ | 19296912 | DSM-IV | AN (n = 36) AN-rec (n = 27) | HCs (n = 44) | ELISA |
| Ehrlich, 2009b^82^ | 18803171 | DSM-IV | AN (n = 32) AN-rec (n = 32) | HCs (n = 32) | ELISA |
| Ehrlich, 2009c^83^ | 19568484 | DSM-IV | AN (n = 33) AN-rec (n = 20) | HCs (n = 33) | ELISA |
| Fernández-García, 2009^84^ | 19302720 | DSM-IV | AN (n = 48) | HCs (n = 20) | RIA |
| Uzum, 2009^85^ | 18803679 | DSM-IV | AN (n = 19) |  | RIA |
| Vögele, 2009^86^ | 19497332 | DSM-IV | BN (n = 17) |  | RIA |
| Arimura, 2010^87^ | 20458162 | DSM-IV | AN (n = 20) | HCs (n = 12) | ELISA |
| Bronsky, 2010^88^ | 21823139 | ICD-10 | AN (n = 36) | HCs (n = 14) | RIA |
| Dostálová, 2010^89^ | 20416056 | DSM-IV | AN (n = 16) | HCs (n = 25) | ELISA |
| Fazeli, 2010a^90^ | 19850693 | DSM-IV | AN (n = 20) | HCs (n = 10) | ELISA |
| Fazeli, 2010b^91^ | 20668047 | DSM-IV | AN (n = 21) |  | RIA |
| Fazeli, 2010c^92^ | 19926712 | DSM-IV | AN (n = 11) | HCs (n = 12) | RIA |
| Haas, 2010^93^ | 20452104 | DSM-IV | AN (n = 73) | HCs (n = 23) | ELISA |
| Lawson, 2010^94^ | 21098684 | DSM-IV | AN (n = 16) | HCs (n = 20) | RIA |
| Legroux-Gérot, 2010^95^ | 20052458 | DSM-IV | AN (n = 103) |  | RIA |
| Nogueira, 2010^96^ | 20485307 | DSM-IV | AN (n = 24) | HCs (n = 14) | ELISA |
| Weiss, 2010^97^ | 20628265 | DSM-IV | AN (n = 29) AN-rec (n = 29) | HCs (n = 29) | ELISA |
| Focker, 2011^98^ | 21258826 | DSM-IV | AN (n = 74) |  | ELISA |
| Smitka, 2011^99^ | 22093818 | DSM-IV | BN (n = 8) | HCs (n = 8) | RIA |
| Fazeli, 2012^100^ | 22508185 | DSM-IV | AN (n = 7) AN-rec (n = 7) | HCs (n = 15) | ELISA |
| Galusca, 2012^101^ | 22023779 | DSM-IV | AN (n = 56) | HCs (n = 42) | ELISA |
| Kavàlkovà, 2012^102^ | 22292722 | DSM-IV | AN (n = 18) | HCs (n = 16) | ELISA |
| Lawson, 2012a^103^ | 22872688 | DSM-IV | AN (n = 13) AN-rec (n = 9) | HCs (n = 13) | RIA |
| Lawson, 2012b^104^ | 21781144 | DSM-IV | AN (n = 15) | HCs (n = 20) | RIA |
| Zepf, 2012^105^ | 22393314 | ICD-10 | AN (n = 14) AN-rec (n = 19) | HCs (n = 15) | RIA |
| Giel, 2013^106^ | 24161801 | DSM-IV | AN (n = 15) | HCs (n = 15) | ELISA |
| Smitka, 2013^107^ | 23318497 | DSM-IV | BN (n = 9) | HCs (n = 9) | RIA |
| Andries, 2014^108^ | 24890912 | DSM-IV-TR | AN (n = 24) |  | RIA |
| Cominato, 2014^109^ | 25322903 | DSM-IV | AN (n = 22) |  | ELISA |
| Pitts, 2014^110^ | 24613094 | DSM-5 | AN (n = 37) |  | RIA |
| Rybakowski, 2014^111^ | 24625915 | DSM-IV-TR | AN (n = 70) |  | ELISA |
| Śmiarowska, 2014^112^ | 24662784 | DSM-IV-TR | AN (n = 63) | HCs (n = 52) | RIA |
| Bodell, 2015^113^ | 26191637 | DSM-5 | BN (n = 32) | HCs (n = 30) | ELISA |
| Conti, 2015^114^ | 25998413 | DSM-5 | AN (n = 24) |  | ELISA |
| Eddy, 2015^115^ | 25098834 | DSM-5 | AN (n = 75) | HCs (n = 22) | ELISA |
| Omodei, 2015^116^ | 25500208 | DSM-5 | AN (n = 15) | HCs (n = 20) | ELISA |
| Andrisani, 2016^117^ | 27910716 | DSM-IV | AN-rec (n = 8) | HCs (n = 10) | RIA |
| Maïmoun, 2016a^118^ | 26418163 | DSM-IV | AN (n = 21) | HCs (n = 19) | ELISA |
| Maïmoun, 2016b^119^ | 26245848 | DSM-IV | AN (n = 50) | HCs (n = 50) | ELISA |
| Seitz, 2016^120^ | 26847072 | DSM-IV | AN (n = 121) |  | ELISA |
| Stroe-Kunold, 2016^121^ | 28030575 | DSM-IV | AN (n = 20) | HCs (n = 25) | ELISA |
| Baranowska-Bik, 2017^122^ | 28604943 | DSM-IV | AN (n = 20) | HCs (n = 27) | RIA |
| Carlsson, 2017^123^ | 28455680 | DSM-IV | AN (n = 20) | HCs (n = 78) | ELISA |
| Estour, 2017^124^ | 28692876 | DSM-IV | AN (n = 40) | HCs (n = 54) | RIA |
| Keel, 2017^125^ | 29044587 | DSM-5 | BN (n = 53) |  | ELISA |
| Maïmoun, 2018^126^ | 29633301 | DSM-5 | AN (n = 286) | HCs (n = 130) | RIA |
| Elegido, 2019^127^ | 30981680 | DSM-5 | AN (n = 41) | HCs (n = 41) | ELISA |
| Lackner, 2019^128^ | 30670293 | ICD-10 | AN (n = 18) |  | ELISA |
| Paslakis, 2019^129^ | 31121263 | DSM-5 | AN (n = 51) | HCs (n = 106) | ELISA |
| Smitka, 2019^130^ | 30915029 | DSM-IV | BN (n = 12) | HCs (n = 12) | RIA |
| Cassioli, 2020^131^ | 32464424 | DSM-5 | AN (n = 26) | HCs (n = 41) | ELISA |
| Mariani, 2020^132^ | 33202604 | DSM-5 | AN (n = 27) | HCs (n = 26) | ELISA |
| Dardennes, 2021^133^ | 33880836 | DSM-IV | AN (n = 26) |  | ELISA |
| Malczyk, 2021^134^ | 34579156 | DSM-5 | AN (n = 31) | HCs (n = 38) | RIA |
| Caldas, 2022^135^ | 35758834 | DSM-5 | BED (n = 13) |  | ELISA |
| Grigioni, 2022^136^ | 35810568 | DSM-IV | AN (n = 17) | HCs (n = 34) | ELISA |
| Tyszkiewicz-Nwafor, 2022^137^ | 34861598 | DSM-5 | AN (n = 17) | HCs (n = 30) | ELISA |
| Amerio, 2023^138^ | 37373917 | DSM-5 | AN (n = 32) | HCs (n = 22) | ELISA |
| Bigazzi, 2023^139^ | 37999906 | DSM-5 | AN (n = 20) | HCs (n = 20) | ELISA |
| Murray Hurtado, 2023^140^ | 37563070 | DSM-5 | AN (n = 30) |  | RIA |
| Schloesser, 2023^141^ | 37319038 | DSM-5 | AN-rec (n = 33) | HCs (n = 36) | ELISA |
| Wronski, 2023a^142^ | 36464660 | DSM-5 | AN (n = 168) | HCs (n = 168) | ELISA |
| Wronski, 2023b^143^ | 37688292 | DSM-5 | AN (n = 44) | HCs (n = 48) | ELISA |
| Wronski, 2023c^144^ | 37797814 | DSM-5 | AN (n = 89) AN-rec (n = 76) | HCs (n = 262) | ELISA |
| Alzaid, 2024^145^ | 38243035 | DSM-IV | AN (n = 78) | HCs (n = 100) | ELISA |
| Bahnsen, 2024^146^ | 38018338 | DSM-5 | AN (n = 142)  AN-rec (n = 43) | HCs (n = 256) | ELISA |

AN, Anorexia Nervosa; AN-rec, Anorexia Nervosa Recovered; BED, Binge-Eating Disorder; BN, Bulimia Nervosa; DSM, Diagnostic and Statistical Manual of Mental Disorders; ED, Eating Disorder; ELISA, Enzyme-Linked Immunosorbent Assay; HCs, Healthy Controls; ICD, International Classification of Diseases; LIFA, Ligand-Mediated Immunofunctional Assay; RIA, Radioimmunoassay

**References**

1. Grinspoon, S. *et al.* Serum leptin levels in women with anorexia nervosa. *J. Clin. Endocrinol. Metab.* **81**, 3861–3863 (1996).

2. Baranowska, B., Wasilewska-Dziubińska, E., Radzikowska, M., Płonowski, A. & Roguski, K. Neuropeptide Y, galanin, and leptin release in obese women and in women with anorexia nervosa. *Metabolism.* **46**, 1384–1389 (1997).

3. Casanueva, F. F. *et al.* Serum immunoreactive leptin concentrations in patients with anorexia nervosa before and after partial weight recovery. *Biochem. Mol. Med.* **60**, 116–120 (1997).

4. Karhunen, L. J., Lappalainen, R. I., Tammela, L., Turpeinen, A. K. & Uusitupa, M. I. Subjective and physiological cephalic phase responses to food in obese binge-eating women. *Int. J. Eat. Disord.* **21**, 321–328 (1997).

5. Mantzoros, C., Flier, J. S., Lesem, M. D., Brewerton, T. D. & Jimerson, D. C. Cerebrospinal fluid leptin in anorexia nervosa: correlation with nutritional status and potential role in resistance to weight gain. *J. Clin. Endocrinol. Metab.* **82**, 1845–1851 (1997).

6. Audi, L. *et al.* Leptin in relation to resumption of menses in women with anorexia nervosa. *Mol. Psychiatry* **3**, 544–547 (1998).

7. Balligand, J., Brichard, S., Brichard, V., Desager, J. & Lambert, M. Hypoleptinemia in patients with anorexia nervosa: loss of circadian rhythm and unresponsiveness to short-term refeeding. *Eur. J. Endocrinol.* **138**, 415–420 (1998).

8. Eckert, E. D. *et al.* Leptin in anorexia nervosa. *J. Clin. Endocrinol. Metab.* **83**, 791–795 (1998).

9. Herpertz, S. *et al.* Circadian plasma leptin levels in patients with anorexia nervosa: relation to insulin and cortisol. *Horm. Res.* **50**, 197–204 (1998).

10. Støving, R. K. *et al.* Diurnal variation of the serum leptin concentration in patients with anorexia nervosa. *Clin. Endocrinol. (Oxf.)* **48**, 761–768 (1998).

11. Ballauff, A. *et al.* Serum leptin and gonadotropin levels in patients with anorexia nervosa during weight gain. *Mol. Psychiatry* **4**, 71–75 (1999).

12. Gendall, K. A., Kaye, W. H., Altemus, M., McConaha, C. W. & La Via, M. C. Leptin, neuropeptide Y, and peptide YY in long-term recovered eating disorder patients. *Biol. Psychiatry* **46**, 292–299 (1999).

13. Haluzík, M. *et al.* Relationship of serum leptin levels and selected nutritional parameters in patients with protein-caloric malnutrition. *Nutr. Burbank Los Angel. Cty. Calif* **15**, 829–833 (1999).

14. Haluzík, M., Papezová, M., Nedvídková, J. & Kábrt, J. Serum leptin levels in patients with anorexia nervosa before and after partial refeeding, relationships to serum lipids and biochemical nutritional parameters. *Physiol. Res.* **48**, 197–202 (1999).

15. Lear, S. A., Pauly, R. P. & Birmingham, C. L. Body fat, caloric intake, and plasma leptin levels in women with anorexia nervosa. *Int. J. Eat. Disord.* **26**, 283–288 (1999).

16. Morgan, J. F. *et al.* Changes in plasma concentrations of leptin and body fat composition during weight restoration in anorexia nervosa. *J. Clin. Endocrinol. Metab.* **84**, 2257 (1999).

17. Nakai, Y., Hamagaki, S., Takagi, R., Taniguchi, A. & Kurimoto, F. Plasma concentrations of tumor necrosis factor-alpha (TNF-alpha) and soluble TNF receptors in patients with anorexia nervosa. *J. Clin. Endocrinol. Metab.* **84**, 1226–1228 (1999).

18. Soyka, L. A., Grinspoon, S., Levitsky, L. L., Herzog, D. B. & Klibanski, A. The effects of anorexia nervosa on bone metabolism in female adolescents. *J. Clin. Endocrinol. Metab.* **84**, 4489–4496 (1999).

19. Støving, R. K. *et al.* Jointly amplified basal and pulsatile growth hormone (GH) secretion and increased process irregularity in women with anorexia nervosa: indirect evidence for disruption of feedback regulation within the GH-insulin-like growth factor I axis. *J. Clin. Endocrinol. Metab.* **84**, 2056–2063 (1999).

20. Baranowska, B., Radzikowska, M., Wasilewska-Dziubinska, E., Roguski, K. & Borowiec, M. Disturbed release of gastrointestinal peptides in anorexia nervosa and in obesity. *Diabetes Obes. Metab.* **2**, 99–103 (2000).

21. Brewerton, T. D., Lesem, M. D., Kennedy, A. & Garvey, W. T. Reduced plasma leptin concentrations in bulimia nervosa. *Psychoneuroendocrinology* **25**, 649–658 (2000).

22. Monteleone, P. *et al.* Plasma leptin response to acute fasting and refeeding in untreated women with bulimia nervosa. *J. Clin. Endocrinol. Metab.* **85**, 2499–2503 (2000).

23. Nedvídková, J., Papezová, H., Haluzík, M. & Schreiber, V. Interaction between serum leptin levels and hypothalamo-hypophyseal-thyroid axis in patients with anorexia nervosa. *Endocr. Res.* **26**, 219–230 (2000).

24. Polito, A. *et al.* Basal metabolic rate in anorexia nervosa: relation to body composition and leptin concentrations. *Am. J. Clin. Nutr.* **71**, 1495–1502 (2000).

25. Baranowska, B., Wolinska-Witort, E., Wasilewska-Dziubinska, E., Roguski, K. & Chmielowska, M. Plasma leptin, neuropeptide Y (NPY) and galanin concentrations in bulimia nervosa and in anorexia nervosa. *Neuro Endocrinol. Lett.* **22**, 356–358 (2001).

26. Adami, G., Campostano, A., Cella, F. & Scopinaro, N. Serum leptin concentration in obese patients with binge eating disorder. *Int. J. Obes.* **26**, 1125–1128 (2002).

27. Adami, G., Campostano, A., Cella, F. & Ferrandes, G. Serum leptin level and restrained eating: study with the Eating Disorder Examination. *Physiol. Behav.* **75**, 189–192 (2002).

28. Heer, M., Mika, C., Grzella, I., Drummer, C. & Herpertz-Dahlmann, B. Changes in bone turnover in patients with anorexia nervosa during eleven weeks of inpatient dietary treatment. *Clin. Chem.* **48**, 754–760 (2002).

29. Krizova, J. *et al.* Soluble leptin receptor levels in patients with anorexia nervosa. *Endocr. Res.* **28**, 199–205 (2002).

30. Monteleone, P., Martiadis, V., Colurcio, B. & Maj, M. Leptin secretion is related to chronicity and severity of the illness in bulimia nervosa. *Psychosom. Med.* **64**, 874–879 (2002).

31. Monteleone, P., Fabrazzo, M., Tortorella, A., Fuschino, A. & Maj, M. Opposite modifications in circulating leptin and soluble leptin receptor across the eating disorder spectrum. *Mol. Psychiatry* **7**, 641–646 (2002).

32. Støving, R. K. *et al.* Indirect evidence for decreased hypothalamic somatostatinergic tone in anorexia nervosa. *Clin. Endocrinol. (Oxf.)* **56**, 391–396 (2002).

33. Brambilla, F. *et al.* Persistent amenorrhoea in weight-recovered anorexics: Psychological and biological aspects. *Psychiatry Res.* **118**, 249–257 (2003).

34. Brown, N. W. *et al.* Evidence for metabolic and endocrine abnormalities in subjects recovered from anorexia nervosa. *Metabolism.* **52**, 296–302 (2003).

35. Calandra, C., Musso, F. & Musso, R. The role of leptin in the etiopathogenesis of anorexia nervosa and bulimia. *Eat. Weight Disord. EWD* **8**, 130–137 (2003).

36. Delporte, M. L., Brichard, S. M., Hermans, M. P., Beguin, C. & Lambert, M. Hyperadiponectinaemia in anorexia nervosa. *Clin. Endocrinol. (Oxf.)* **58**, 22–29 (2003).

37. Holtkamp, K. *et al.* Elevated physical activity and low leptin levels co-occur in patients with anorexia nervosa. *J. Clin. Endocrinol. Metab.* **88**, 5169–5174 (2003).

38. Holtkamp, K. *et al.* The effect of therapeutically induced weight gain on plasma leptin levels in patients with anorexia nervosa. *J. Psychiatr. Res.* **37**, 165–169 (2003).

39. Krsek, M. *et al.* Plasma ghrelin levels and malnutrition: a comparison of two etiologies. *Eat. Weight Disord. EWD* **8**, 207–211 (2003).

40. Lindberg, N. *et al.* Growth hormone-insulin-like growth factor-1 axis, leptin and sleep in anorexia nervosa patients. *Neuropsychobiology* **47**, 78–85 (2003).

41. Lob, S. *et al.* Serum Leptin Monitoring in Anorectic Patients During Refeeding Therapy. *Exp. Clin. Endocrinol. Diabetes* **111**, 278–282 (2003).

42. Misra, M. *et al.* Serum osteoprotegerin in adolescent girls with anorexia nervosa. *J. Clin. Endocrinol. Metab.* **88**, 3816–3822 (2003).

43. Pannacciulli, N. *et al.* Anorexia nervosa is characterized by increased adiponectin plasma levels and reduced nonoxidative glucose metabolism. *J. Clin. Endocrinol. Metab.* **88**, 1748–1752 (2003).

44. Weinbrenner, T., Zittermann, A., Gouni-Berthold, I., Stehle, P. & Berthold, H. K. Body mass index and disease duration are predictors of disturbed bone turnover in anorexia nervosa. A case-control study. *Eur. J. Clin. Nutr.* **57**, 1262–1267 (2003).

45. Holtkamp, K. *et al.* High serum leptin levels subsequent to weight gain predict renewed weight loss in patients with anorexia nervosa. *Psychoneuroendocrinology* **29**, 791–797 (2004).

46. Miller, K. K. *et al.* Preservation of neuroendocrine control of reproductive function despite severe undernutrition. *J. Clin. Endocrinol. Metab.* **89**, 4434–4438 (2004).

47. Misra, M. *et al.* Hormonal and body composition predictors of soluble leptin receptor, leptin, and free leptin index in adolescent girls with anorexia nervosa and controls and relation to insulin sensitivity. *J. Clin. Endocrinol. Metab.* **89**, 3486–3495 (2004).

48. Tagami, T. *et al.* Adiponectin in anorexia nervosa and bulimia nervosa. *J. Clin. Endocrinol. Metab.* **89**, 1833–1837 (2004).

49. Bosy-Westphal, A. *et al.* Determinants of plasma adiponectin levels in patients with anorexia nervosa examined before and after weight gain. *Eur. J. Nutr.* **44**, 355–359 (2005).

50. Dostálová, I. *et al.* Leptin concentrations in the abdominal subcutaneous adipose tissue of patients with anorexia nervosa assessed by in vivo microdialysis. *Regul. Pept.* **128**, 63–68 (2005).

51. Haas, V. *et al.* Leptin and body weight regulation in patients with anorexia nervosa before and during weight recovery. *Am. J. Clin. Nutr.* **81**, 889–896 (2005).

52. Housova, J. *et al.* Serum adiponectin and resistin concentrations in patients with restrictive and binge/purge form of anorexia nervosa and bulimia nervosa. *J. Clin. Endocrinol. Metab.* **90**, 1366–1370 (2005).

53. Manara, F., Manara, A. & Todisco, P. Correlation between psychometric and biological parameters in anorexic and bulimic patients during and after an intensive day hospital treatment. *Eat. Weight Disord. EWD* **10**, 236–244 (2005).

54. Monteleone, P. *et al.* Blood levels of the endocannabinoid anandamide are increased in anorexia nervosa and in binge-eating disorder, but not in bulimia nervosa. *Neuropsychopharmacol. Off. Publ. Am. Coll. Neuropsychopharmacol.* **30**, 1216–1221 (2005).

55. Oświecimska, J., Ziora, K., Geisler, G. & Broll-Waśka, K. Prospective evaluation of leptin and neuropeptide Y (NPY) serum levels in girls with anorexia nervosa. *Neuro Endocrinol. Lett.* **26**, 301–304 (2005).

56. Shimizu, T. *et al.* Factors involved in the regulation of plasma leptin levels in children and adolescents with anorexia nervosa. *Pediatr. Int. Off. J. Jpn. Pediatr. Soc.* **47**, 154–158 (2005).

57. Bossu, C. *et al.* Energy expenditure adjusted for body composition differentiates constitutional thinness from both normal subjects and anorexia nervosa. *Am. J. Physiol. Endocrinol. Metab.* **292**, E132-137 (2007).

58. Dostalova, I., Kunesova, M., Duskova, J., Papezova, H. & Nedvidkova, J. Adipose tissue resistin levels in patients with anorexia nervosa. *Nutr. Burbank Los Angel. Cty. Calif* **22**, 977–983 (2006).

59. Holtkamp, K. *et al.* Physical Activity and Restlessness Correlate with Leptin Levels in Patients with Adolescent Anorexia Nervosa. *Biol. Psychiatry* **60**, 311–313 (2006).

60. Miljic, D. *et al.* Ghrelin has partial or no effect on appetite, growth hormone, prolactin, and cortisol release in patients with anorexia nervosa. *J. Clin. Endocrinol. Metab.* **91**, 1491–1495 (2006).

61. Misra, M. *et al.* Nutrient intake in community-dwelling adolescent girls with anorexia nervosa and in healthy adolescents. *Am. J. Clin. Nutr.* **84**, 698–706 (2006).

62. Misra, M. *et al.* Role of cortisol in menstrual recovery in adolescent girls with anorexia nervosa. *Pediatr. Res.* **59**, 598–603 (2006).

63. Tanaka, M. *et al.* Ghrelin concentrations and cardiac vagal tone are decreased after pharmacologic and cognitive-behavioral treatment in patients with bulimia nervosa. *Horm. Behav.* **50**, 261–265 (2006).

64. Brambilla, F., Monteleone, P. & Maj, M. Olanzapine-induced weight gain in anorexia nervosa: Involvement of leptin and ghrelin secretion? *Psychoneuroendocrinology* **32**, 402–406 (2007).

65. Dolezalova, R. *et al.* Changes of endocrine function of adipose tissue in anorexia nervosa: comparison of circulating levels versus subcutaneous mRNA expression. *Clin. Endocrinol. (Oxf.)* **67**, 674–678 (2007).

66. Dostalova, I., Bartak, V., Papezova, H. & Nedvidkova, J. The effect of short-term exercise on plasma leptin levels in patients with anorexia nervosa. *Metabolism.* **56**, 497–503 (2007).

67. Dostálová, I., Smitka, K., Papežová, H., Kvasnicková, H. & Nedvídková, J. Increased insulin sensitivity in patients with anorexia nervosa: the role of adipocytokines. *Physiol. Res.* **56**, 587–594 (2007).

68. Germain, N. *et al.* Constitutional thinness and lean anorexia nervosa display opposite concentrations of peptide YY, glucagon-like peptide 1, ghrelin, and leptin. *Am. J. Clin. Nutr.* **85**, 967–971 (2007).

69. Ohwada, R., Hotta, M., Sato, K., Shibasaki, T. & Takano, K. The relationship between serum levels of estradiol and osteoprotegerin in patients with anorexia nervosa. *Endocr. J.* **54**, 953–959 (2007).

70. Dostálová, I. *et al.* Plasma concentrations of fibroblast growth factors 19 and 21 in patients with anorexia nervosa. *J. Clin. Endocrinol. Metab.* **93**, 3627–3632 (2008).

71. Dynesen, A. W. *et al.* Meal-induced compositional changes in blood and saliva in persons with bulimia nervosa. *Am. J. Clin. Nutr.* **87**, 12–22 (2008).

72. Ehrlich, S. *et al.* Platelet monoamine oxidase activity in underweight and weight-recovered females with anorexia nervosa. *Pharmacopsychiatry* **41**, 226–231 (2008).

73. Ehrlich, S. *et al.* S100B in underweight and weight-recovered patients with anorexia nervosa. *Psychoneuroendocrinology* **33**, 782–788 (2008).

74. Ehrlich, S. *et al.* Glial and neuronal damage markers in patients with anorexia nervosa. *J. Neural Transm. Vienna Austria 1996* **115**, 921–927 (2008).

75. Guerdjikova, A. I. *et al.* High-dose escitalopram in the treatment of binge-eating disorder with obesity: a placebo-controlled monotherapy trial. *Hum. Psychopharmacol.* **23**, 1–11 (2008).

76. Holtkamp, K. *et al.* Serum levels of S100B are decreased in chronic starvation and normalize with weight gain. *J. Neural Transm. Vienna Austria 1996* **115**, 937–940 (2008).

77. Misra, M. *et al.* Percentage extremity fat, but not percentage trunk fat, is lower in adolescent boys with anorexia nervosa than in healthy adolescents. *Am. J. Clin. Nutr.* **88**, 1478–1484 (2008).

78. Misra, M. *et al.* Prognostic indicators of changes in bone density measures in adolescent girls with anorexia nervosa-II. *J. Clin. Endocrinol. Metab.* **93**, 1292–1297 (2008).

79. Nakahara, T. *et al.* Plasma obestatin concentrations are negatively correlated with body mass index, insulin resistance index, and plasma leptin concentrations in obesity and anorexia nervosa. *Biol. Psychiatry* **64**, 252–255 (2008).

80. Dostálová, I., Sedláčková, D., Papežová, H., Nedvídková, J. & Haluzík, M. Serum visfatin levels in patients with anorexia nervosa and bulimia nervosa. *Physiol. Res.* **58**, 903–907 (2009).

81. Ehrlich, S. *et al.* The role of leptin and cortisol in hyperactivity in patients with acute and weight-recovered anorexia nervosa. *Prog. Neuropsychopharmacol. Biol. Psychiatry* **33**, 658–662 (2009).

82. Ehrlich, S. *et al.* Aromatic amino acids in weight-recovered females with anorexia nervosa. *Int. J. Eat. Disord.* **42**, 166–172 (2009).

83. Ehrlich, S. *et al.* Serum brain-derived neurotrophic factor and peripheral indicators of the serotonin system in underweight and weight-recovered adolescent girls and women with anorexia nervosa. *J. Psychiatry Neurosci. JPN* **34**, 323–329 (2009).

84. Fernández-García, D. *et al.* Thin healthy women have a similar low bone mass to women with anorexia nervosa. *Br. J. Nutr.* **102**, 709–714 (2009).

85. Uzum, A. K., Yucel, B., Omer, B., Issever, H. & Ozbey, N. C. Leptin concentration indexed to fat mass is increased in untreated anorexia nervosa (AN) patients. *Clin. Endocrinol. (Oxf.)* **71**, 33–39 (2009).

86. Vögele, C., Hilbert, A. & Tuschen-Caffier, B. Dietary restriction, cardiac autonomic regulation and stress reactivity in bulimic women. *Physiol. Behav.* **98**, 229–234 (2009).

87. Arimura, C. *et al.* Predictors of menstrual resumption by patients with anorexia nervosa. *Eat. Weight Disord.* **15**, e226-33 (2010).

88. Bronsky, J. *et al.* Changes of orexin A plasma levels in girls with anorexia nervosa during eight weeks of realimentation. *Int. J. Eat. Disord.* **44**, 547–552 (2011).

89. Dostálová, I. *et al.* Association of macrophage inhibitory cytokine-1 with nutritional status, body composition and bone mineral density in patients with anorexia nervosa: the influence of partial realimentation. *Nutr. Metab.* **7**, 34 (2010).

90. Fazeli, P. K. *et al.* Preadipocyte factor-1 is associated with marrow adiposity and bone mineral density in women with anorexia nervosa. *J. Clin. Endocrinol. Metab.* **95**, 407–413 (2010).

91. Fazeli, P. K. *et al.* Effects of recombinant human growth hormone in anorexia nervosa: a randomized, placebo-controlled study. *J. Clin. Endocrinol. Metab.* **95**, 4889–4897 (2010).

92. Fazeli, P. K., Misra, M., Goldstein, M., Miller, K. K. & Klibanski, A. Fibroblast growth factor-21 may mediate growth hormone resistance in anorexia nervosa. *J. Clin. Endocrinol. Metab.* **95**, 369–374 (2010).

93. Haas, V. K., Gaskin, K. J., Kohn, M. R., Clarke, S. D. & Müller, M. J. Different thermic effects of leptin in adolescent females with varying body fat content. *Clin. Nutr. Edinb. Scotl.* **29**, 639–645 (2010).

94. Lawson, E. A. *et al.* Appetite-regulating hormones cortisol and peptide YY are associated with disordered eating psychopathology, independent of body mass index. *Eur. J. Endocrinol.* **164**, 253–261 (2011).

95. Legroux-Gérot, I. *et al.* Anorexia nervosa, osteoporosis and circulating leptin: the missing link. *Osteoporos. Int. J. Establ. Result Coop. Eur. Found. Osteoporos. Natl. Osteoporos. Found. USA* **21**, 1715–1722 (2010).

96. Nogueira, J.-P. *et al.* Specific adipocytokines profiles in patients with hyperactive and/or binge/purge form of anorexia nervosa. *Eur. J. Clin. Nutr.* **64**, 840–844 (2010).

97. Weiss, D. *et al.* Preproenkephalin expression in peripheral blood mononuclear cells of acutely underweight and recovered patients with anorexia nervosa. *Neuropsychobiology* **62**, 151–157 (2010).

98. Föcker, M. *et al.* Screening for anorexia nervosa via measurement of serum leptin levels. *J. Neural Transm. Vienna Austria 1996* **118**, 571–578 (2011).

99. Smitka, K. *et al.* A higher response of plasma neuropeptide Y, growth hormone, leptin levels and extracellular glycerol levels in subcutaneous abdominal adipose tissue to Acipimox during exercise in patients with bulimia nervosa: single-blind, randomized, microdialysis study. *Nutr. Metab.* **8**, 81 (2011).

100. Fazeli, P. K. *et al.* Marrow fat and preadipocyte factor-1 levels decrease with recovery in women with anorexia nervosa. *J. Bone Miner. Res. Off. J. Am. Soc. Bone Miner. Res.* **27**, 1864–1871 (2012).

101. Galusca, B. *et al.* Normal inhibin B levels suggest partial preservation of gonadal function in adult male patients with anorexia nervosa. *J. Sex. Med.* **9**, 1442–1447 (2012).

102. Kaválková, P. *et al.* Preadipocyte factor-1 concentrations in patients with anorexia nervosa: the influence of partial realimentation. *Physiol. Res.* **61**, 153–159 (2012).

103. Lawson, E. A. *et al.* Oxytocin secretion is associated with severity of disordered eating psychopathology and insular cortex hypoactivation in anorexia nervosa. *J. Clin. Endocrinol. Metab.* **97**, E1898-1908 (2012).

104. Lawson, E. A. *et al.* Leptin levels are associated with decreased depressive symptoms in women across the weight spectrum, independent of body fat. *Clin. Endocrinol. (Oxf.)* **76**, 520–525 (2012).

105. Zepf, F. D. *et al.* Differences in zinc status and the leptin axis in anorexic and recovered adolescents and young adults: a pilot study. *Food Nutr. Res.* **56**, (2012).

106. Giel, K. E. *et al.* Understanding the reward system functioning in anorexia nervosa: crucial role of physical activity. *Biol. Psychol.* **94**, 575–581 (2013).

107. Smitka, K. *et al.* Short-term exercise combined with Acipimox administration induces an increase in plasma ACTH and its subsequent fall in the recovery phase in bulimic women. *Regul. Pept.* **182**, 45–52 (2013).

108. Andries, A., Gram, B. & Støving, R. K. Effect of dronabinol therapy on physical activity in anorexia nervosa: a randomised, controlled trial. *Eat. Weight Disord. EWD* **20**, 13–21 (2015).

109. Cominato, L. *et al.* Menstrual cycle recovery in patients with anorexia nervosa: the importance of insulin-like growth factor 1. *Horm. Res. Paediatr.* **82**, 319–323 (2014).

110. Pitts, S., Blood, E., Divasta, A. & Gordon, C. M. Percentage body fat by dual-energy X-ray absorptiometry is associated with menstrual recovery in adolescents with anorexia nervosa. *J. Adolesc. Health Off. Publ. Soc. Adolesc. Med.* **54**, 739–741 (2014).

111. Rybakowski, F., Slopien, A. & Tyszkiewicz-Nwafor, M. Inverse relationship between leptin increase and improvement in depressive symptoms in anorexia nervosa. *Neuro Endocrinol. Lett.* **35**, 64–67 (2014).

112. Śmiarowska, M. *et al.* Association of plasma hormones, nutritional status, and stressful life events in anorexia nervosa patients. *Postepy Hig. Med. Doswiadczalnej Online* **68**, 162–171 (2014).

113. Bodell, L. P. & Keel, P. K. Weight suppression in bulimia nervosa: Associations with biology and behavior. *J. Abnorm. Psychol.* **124**, 994–1002 (2015).

114. Conti, E. *et al.* Beta-amyloid plasma levels in adolescents with anorexia nervosa of the restrictive type. *Neuropsychobiology* **71**, 154–157 (2015).

115. Eddy, K. T. *et al.* Appetite regulatory hormones in women with anorexia nervosa: binge-eating/purging versus restricting type. *J. Clin. Psychiatry* **76**, 19–24 (2015).

116. Omodei, D. *et al.* Immune-metabolic profiling of anorexic patients reveals an anti-oxidant and anti-inflammatory phenotype. *Metabolism.* **64**, 396–405 (2015).

117. Andrisani, A. *et al.* Persistent amenorrhea and decreased DHEAS to cortisol ratio after recovery from anorexia nervosa. *Gynecol. Endocrinol. Off. J. Int. Soc. Gynecol. Endocrinol.* **33**, 311–314 (2017).

118. Maïmoun, L. *et al.* Is Serum Serotonin Involved in the Bone Loss of Young Females with Anorexia Nervosa? *Horm. Metab. Res. Horm. Stoffwechselforschung Horm. Metab.* **48**, 174–177 (2016).

119. Maïmoun, L. *et al.* Evidence of a link between resting energy expenditure and bone remodelling, glucose homeostasis and adipokine variations in adolescent girls with anorexia nervosa. *Osteoporos. Int. J. Establ. Result Coop. Eur. Found. Osteoporos. Natl. Osteoporos. Found. USA* **27**, 135–146 (2016).

120. Seitz, J. *et al.* Leptin levels in patients with anorexia nervosa following day/inpatient treatment do not predict weight 1 year post-referral. *Eur. Child Adolesc. Psychiatry* **25**, 1019–1025 (2016).

121. Stroe-Kunold, E. *et al.* Time Course of Leptin in Patients with Anorexia Nervosa during Inpatient Treatment: Longitudinal Relationships to BMI and Psychological Factors. *PloS One* **11**, e0166843 (2016).

122. Baranowska-Bik, A. *et al.* Adipokine profile in patients with anorexia nervosa. *Endokrynol. Pol.* **68**, 422–429 (2017).

123. Carlsson, M., Brudin, L. & Wanby, P. Directly measured free 25-hydroxy vitamin D levels show no evidence of vitamin D deficiency in young Swedish women with anorexia nervosa. *Eat. Weight Disord. EWD* **23**, 247–254 (2018).

124. Estour, B. *et al.* Differentiating constitutional thinness from anorexia nervosa in DSM 5 era. *Psychoneuroendocrinology* **84**, 94–100 (2017).

125. Keel, P. K., Bodell, L. P., Haedt-Matt, A. A., Williams, D. L. & Appelbaum, J. Weight suppression and bulimic syndrome maintenance: Preliminary findings for the mediating role of leptin. *Int. J. Eat. Disord.* **50**, 1432–1436 (2017).

126. Maïmoun, L. *et al.* Effects of the two types of anorexia nervosa (binge eating/purging and restrictive) on bone metabolism in female patients. *Clin. Endocrinol. (Oxf.)* **88**, 863–872 (2018).

127. Elegido, A. *et al.* Adipokines, cortisol and cytokine alterations in recent onset anorexia nervosa. A case-control study. *Endocrinol. Diabetes Nutr.* **66**, 571–578 (2019).

128. Lackner, S. *et al.* Novel approaches for the assessment of relative body weight and body fat in diagnosis and treatment of anorexia nervosa: A cross-sectional study. *Clin. Nutr. Edinb. Scotl.* **38**, 2913–2921 (2019).

129. Paslakis, G. *et al.* Associations between neuropsychological performance and appetite-regulating hormones in anorexia nervosa and healthy controls: Ghrelin’s putative role as a mediator of decision-making. *Mol. Cell. Endocrinol.* **497**, 110441 (2019).

130. Smitka, K. *et al.* Acipimox Administration With Exercise Induces a Co-feedback Action of the GH, PP, and PYY on Ghrelin Associated With a Reduction of Peripheral Lipolysis in Bulimic and Healthy-Weight Czech Women: A Randomized Study. *Front. Endocrinol.* **10**, 108 (2019).

131. Cassioli, E. *et al.* Reward and psychopathological correlates of eating disorders: The explanatory role of leptin. *Psychiatry Res.* **290**, 113071 (2020).

132. Mariani, S. *et al.* Blood SIRT1 Shows a Coherent Association with Leptin and Adiponectin in Relation to the Degree and Distribution of Adiposity: A Study in Obesity, Normal Weight and Anorexia Nervosa. *Nutrients* **12**, 3506 (2020).

133. Dardennes, R. *et al.* Lower leptin level at discharge in acute anorexia nervosa is associated with early weight-loss. *Eur. Eat. Disord. Rev. J. Eat. Disord. Assoc.* **29**, 634–644 (2021).

134. Malczyk, Ż. *et al.* Exocrine Pancreatic Function in Girls with Anorexia Nervosa. *Nutrients* **13**, 3280 (2021).

135. do Rosário Caldas, N. *et al.* Binge eating disorder, frequency of depression, and systemic inflammatory state in individuals with obesity - A cross sectional study. *Arch. Endocrinol. Metab.* **66**, 489–497 (2022).

136. Grigioni, S. *et al.* Intestinal permeability and appetite regulating peptides-reactive immunoglobulins in severely malnourished women with anorexia nervosa. *Clin. Nutr. Edinb. Scotl.* **41**, 1752–1758 (2022).

137. Tyszkiewicz-Nwafor, M. *et al.* Expression of immune-related proteins and their association with neuropeptides in adolescent patients with anorexia nervosa. *Neuropeptides* **91**, 102214 (2022).

138. Amerio, A. *et al.* The Association between Blood SIRT1 and Ghrelin, Leptin, and Antibody Anti-Hypothalamus: A Comparison in Normal Weight and Anorexia Nervosa. *J. Pers. Med.* **13**, 928 (2023).

139. Bigazzi, F. *et al.* PCSK9 and leptin plasma levels in anorexia nervosa. *Horm. Athens Greece* **23**, 137–140 (2024).

140. Murray Hurtado, M., Martín Rivada, Á., Quintero Alemán, C., Ruiz Alcántara, M. P. & Ramallo Fariña, Y. Body composition and nutritional status changes in adolescents with anorexia nervosa. *An. Pediatr.* **99**, 162–169 (2023).

141. Schloesser, L. *et al.* Sex-dependent clinical presentation, body image, and endocrine status in long-term remitted anorexia nervosa. *Eur. Eat. Disord. Rev. J. Eat. Disord. Assoc.* **31**, 696–708 (2023).

142. Wronski, M.-L. *et al.* Differential alterations of amygdala nuclei volumes in acutely ill patients with anorexia nervosa and their associations with leptin levels. *Psychol. Med.* **53**, 6288–6303 (2023).

143. Wronski, M.-L. *et al.* Explicating the role of amygdala substructure alterations in the link between hypoleptinemia and rumination in anorexia nervosa. *Acta Psychiatr. Scand.* **148**, 368–381 (2023).

144. Wronski, M.-L. *et al.* Dynamic Amygdala Nuclei Alterations in Relation to Weight Status in Anorexia Nervosa Are Mediated by Leptin. *J. Am. Acad. Child Adolesc. Psychiatry* **63**, 624–639 (2024).

145. Alzaid, H. *et al.* Hypothalamic subregion alterations in anorexia nervosa and obesity: Association with appetite-regulating hormone levels. *Int. J. Eat. Disord.* **57**, 581–592 (2024).

146. Bahnsen, K. *et al.* Differential longitudinal changes of hippocampal subfields in patients with anorexia nervosa. *Psychiatry Clin. Neurosci.* **78**, 186–196 (2024).
